# T-Cell Metabolism in Hematopoietic Cell Transplantation

**DOI:** 10.3389/fimmu.2018.00176

**Published:** 2018-02-09

**Authors:** Hung D. Nguyen, Sandeepkumar Kuril, David Bastian, Xue-Zhong Yu

**Affiliations:** ^1^Department of Microbiology and Immunology, Medical University of South Carolina, Charleston, SC, United States; ^2^Department of Pediatric Ematology-Oncology, Medical University of South Carolina, Charleston, SC, United States; ^3^Department of Medicine, Medical University of South Carolina, Charleston, SC, United States

**Keywords:** T cell, metabolism, hematopoietic stem cell transplantation, graft-versus-host disease, glycolysis

## Abstract

Metabolism, including catabolism and anabolism, is a basic cellular process necessary for cell survival. T lymphocytes have a distinct metabolism that can determine both fate and function. T-cell activation depends on glycolysis to obtain materials and energy for proliferation and effector function. Importantly, T cells utilize different metabolic processes under different conditions and diseases. Allogeneic hematopoietic cell transplantation (allo-HCT) is a classic immunotherapy for hematological malignancies; however, the development of graft-versus-host disease (GVHD) is a major factor limiting the success of allo-HCT. T cells in the donor graft drive GVHD by mounting a robust immunological attack against recipient normal tissues. Hence, understanding T-cell metabolism after allo-HCT would provide potential metabolic targets for the control of GVHD and primary tumor relapse. The purpose of the current review is to highlight the key metabolic pathways involved in alloantigen-activated T cells and to discuss how manipulating these pathways can serve as potential new therapeutic strategies to induce immune tolerance after allo-transplantation. We will also summarize the recent progress in regulating T-cell metabolism in bone marrow transplantation by targeting novel metabolic regulators or immune checkpoint molecules.

## Introduction

Allogeneic bone marrow transplantation [BMT; allogeneic hematopoietic stem-cell transplantation (allo-HCT)] is a curative option to treat hematological malignancies. However, graft-versus-host disease (GVHD) limits the success of allo-HCT (1). GVHD pathogenesis is characterized by a robust immunological attack by donor T cells against normal tissues of transplanted recipients (2). As donor T cells are the driving force in GVHD, suppressing T-cell responses is a standard therapeutic approach for the treatment of GVHD. However, these broadly immunosuppressive drugs, including corticosteroids and inhibitors of calcineurin or mammalian target of rapamycin (mTOR), leave patients highly susceptible to infections and induce remission in <50% of patients. The mortality rate of patients with steroid-refractory aGVHD is close to 90% (3). Hence, understanding T-cell pathobiology is critical to the development of effective therapies to prevent GVHD. Cell metabolism impacts the fate and function of T cells (4). Targeting T-cell metabolism is a viable therapeutic strategy in other immunological disorders, including systemic lupus erythematosus, rheumatoid arthritis, and experimental autoimmune encephalomyelitis (5–7). A growing body of evidence from multiple studies suggests T-cell metabolism is a promising target for controlling GVHD. Recently, our group and others attempted to characterize the metabolic profile of donor T cells following allo-HCT, yet a consensus on the data has not been reached (2, 8, 9). In this review, we will detail the recent findings in the evolving field of immuno-metabolism with a focus on T-cell metabolism in the context of allo-HCT and discuss how this knowledge can help us reevaluate our current understanding of immune activation and suppression after allo-HCT, and promising immunotherapeutic strategies to archive long-term transplantation tolerance in transplanted recipients aiming to prevent allograft rejection and GVHD.

## Overview of T-Cell Metabolism

Glycolysis and oxidative phosphorylation (OXPHOS) are fundamental cellular processes in generating energy, or adenosine triphosphate (ATP) (10, 11). Naïve T cells rely primarily on OXPHOS to meet their energy demands (12). Upon antigen recognition, naïve T cells clonally expand into T effector cells (Teffs). Upon antigen clearance, most of these effector T cells die, but a subset of long-lived memory T cells (Tm) persist with an enhanced mitochondrial capacity relying on fatty acid oxidation (FAO) to fuel OXPHOS (13). OXPHOS can generate up to 36 molecules of ATP. The transition from resting naïve T-cells into activated Teffs requires substantial metabolic reprogramming (12, 13). A Teff’s metabolic profile is characterized by a shift to aerobic glycolysis as a main energy source (12, 14). Aerobic glycolysis involves the mitochondrion-independent metabolism of glucose into pyruvate and provides only two molecules of ATP per glucose (15). While glycolysis is less efficient than OXPHOS at yielding an abundance of ATP per molecule of glucose, aerobic glycolysis supplies metabolic intermediates for cell growth and proliferation as well as induces the pentose phosphate pathway (PPP), which produces nucleotides and amino acids that subsequently generate reducing power in the form of NADH to maintain cellular redox balance (NAD^+^/NADH) (15). Teffs also use glutamine as a carbon source to fuel the tricarboxylic acid (TCA) cycle *via* α-ketoglutarate (α-KG) through the process of glutaminolysis (16, 17).

## Metabolism and CD4^+^T Cell Differentiation

Depending on the nature of antigen and cytokine signal, CD4^+^ T cells differentiate into Th1, Th2, Th9, Th17, T follicular helper cells (Tfh), Tr-1, or Treg. While Th1, Th2, and Th17 are pathogenic, Tr-1 and Treg are suppressive in acute GVHD (18–20). Metabolism plays a critical role in CD4^+^ T-cell differentiation (12). While Th1, Th2, and Th17 lineages preferentially use glycolysis to meet energetic demand though activation of PI3K/Akt/mTOR pathway, CD4^+^ Tregs use mitochondrial-dependent FAO (4). Therefore, enhanced FAO *via* inhibiting mTOR leads to increased Treg generation (21). Hypoxia-inducible factor 1 is the key regulator of anabolic metabolism in Th17 cells (22). Meanwhile, Tfh, a pathogenic T-cell subset in chronic GVHD, depend on glycolysis and lipogenesis to meet energy demands required for differentiation (23). The metabolic profiles of Th9 and Tr1 remain unclear.

## Metabolism of Allogeneic T Cells

### Glucose Metabolism

**Figure 1:**
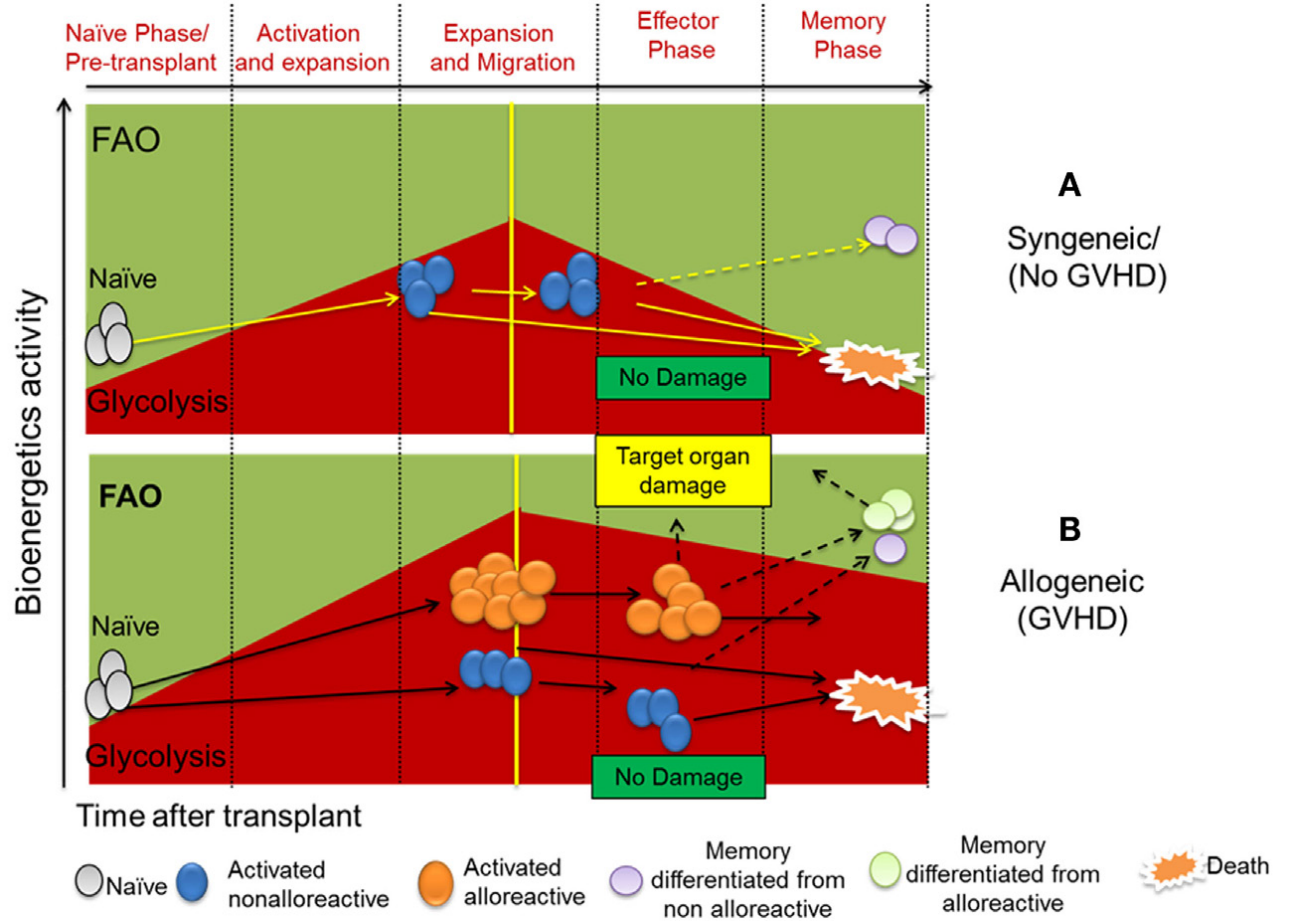
**(A)** Naïve/resting T cells are dependent on oxidative phosphorylation with fatty acid oxidation (FAO) as a major material resource. Upon activation by self-antigens under homeostatic state, naïve/resting T cells reprogram their metabolic phenotype to become partially activated T cells (29), which possess glycolytic metabolic phenotype. Due to lack of specific TCR stimulation, a large proportion of non-alloreactive T cells gradually die. However, specific self-epitopes of T cells can become memory T cells (Tm) which depend upon FAO for their metabolism. **(B)** Upon activation by alloantigen in transplant recipients, naïve/resting T cells proliferate and their memory differentiate to activate T cells both alloreactive and non-alloreactive. Alloreactive T cells and their differentiated memory cells are capable of causing target organ damage. Alloreactive T cells have much higher glycolytic activity compared to non-alloreactive counterpart. Both alloreactive and non-alloreactive T cells can die or differentiate into Tms accordingly. Glucose retention and glycolytic activity decide survival and alloreactivity of alloreactive T cells to induce graft-versus-host disease (GVHD) after allogeneic hematopoietic cell transplantation.

Using MHC-mismatched or haploidentical murine models of BMT, we uncovered that upon alloantigen activation, donor T cells increase both glycolysis and OXPHOS to obtain energetic materials necessary for activation and proliferation (2, 9). Albeit, they preferentially rely on glycolysis to maintain their capacity to induce GVHD (2, 9, 24). While OXPHOS of donor T cells isolated from syngeneic (no GVHD) and allogeneic (GVHD) recipients were similar, the glycolytic activity of donor T cells was significantly higher in allogeneic than syngeneic recipients, indicating an escalation of T-cell glucose metabolism correlated with GVHD development (2) (Figure 1). Furthermore, T cells isolated from livers of allogeneic recipients exhibited higher glycolytic activity compared to those of syngeneic recipients 14 days after allo-HCT, implying an enduring glycolytic response by allogeneic T cells in GVHD target organs. While *in vitro* activated T cells upregulate and maintain expression of Glut1 for sufficient glucose uptake (17), allo-activated T cells also increase Glut 3 to fulfill their extremely high demand for glucose (2). In addition, alloantigen-activated T cells upregulate both hexokinase 1 (HK1) and HK2 to facilitate induction of glycolysis (2). To maintain sufficient glycolytic activity, allogeneic CD4^+^ T cells activate mTOR and increase differentiation into Th1 and Th17 (2, 25) while decreasing Treg generation (24). Inhibition of glycolysis by genetic depletion or pharmacological blockade of mTORC1 (2, 26) or glycolytic checkpoints, including glut-1 (24), HK-2, PFKB3 (2), or PKM2 (unpublished study), reduces alloreactive T-cell generation and subsequently ameliorates GVHD severity. Alternatively, enhancing FAO to inhibit mTOR using PI3K/AKT or AMPK inhibitors (27, 28) effectively prevents GVHD development.

### OXPHOS and Oxidative Stress in Allogeneic T Cells

Allogeneic T cells in lymphoid or target organs of recipients significantly increase OXPHOS compared to resting T cells after allo-HCT (2, 9). Since OXPHOS activity was comparable in allogeneic and syngeneic T cells (2), increased OXPHOS may not be a direct mechanism by which pathogenic T cells are generated. However, due to increased non-mitochondrial oxygen consumption rate (OCR), allogeneic T cells had higher levels of oxidative stress yet lower levels of antioxidants (2, 9). As reactive oxygen species (ROS) are required for T-cell activation (30), this indicates chronic allo-activation of donor T cells after transplant. Increased ROS generation in allogeneic T cells may be the result of a hyperpolarized mitochondrial membrane potential (ΔΨm), subsequently making alloreactive CD4^+^ and CD8^+^T cells highly susceptible to small-molecule inhibitors of mitochondrial F1F0 adenosine triphosphate synthase in haploidentical BMT model (9, 31).

### The Pentose Phosphate Pathway

In murine models of GVHD, alloantigen-activated T cells have increased PPP activity (2, 31). Intracellular glucose metabolized by HK forms glucose 6-phospate (G-6P), which then enters the PPP to generate ribose-5 phosphate (R-5P); the carbon donor during nucleotide biogenesis (32). The conversion of G-6P to R-5P is regulated by glucose-6-phosphate dehydrogenase in the oxidative arm of the PPP (33), which is significantly increased in allogeneic T cells (2, 31). The oxidative arm of the PPP is crucial for the formation of NADPH, which plays a critical role in reductive biosynthesis of antioxidant molecules, such as GSH (34). GSH promotes T-cell expansion by driving glycolysis and glutaminolysis, and supporting mTORC1 and c-Myc signaling in inflammation (35). Due to chronic stimulation by alloantigens, nucleotide biosynthesis is sustained to support anabolic growth of T cells during allogeneic responses; leading to a deficit in purine and pyrimidine catabolism (2) and exhaustion of GS and GSH (9).

### Glutamine Metabolism

Glutamine uptake and metabolism are crucial for normal T-cell function (36). Donor T cells require the rapid synthesis of macromolecules for their growth, proliferation, and for energy after allo-HCT (11). Glutamine converted to glutamate can support the progression of the TCA cycle, ultimately leading to production of α-KG, a citrate precursor. To generate new lipids, citrate is secreted into the cytosol and metabolized to form acetyl-CoA, the backbone for lipid synthesis (34). In addition to the PPP, glutaminolysis can provide NADPH to support lipid and nucleotide biosynthesis as well as maintenance of GSH (37). *In vitro*-activated T cells utilize the transcription factor Myc to incorporate glutamine into metabolic pathways (17). Allogeneic T cells increase glutamine uptake by upregulating glutamine transport channels, such as glutamine-fructose-6-phosphate transaminase, phosphoribosyl pyrophosphate amidotransferase, and glutaminase 2 post allo-HCT (2). While the level of glutamine was increased in allogeneic T cells, the level of glutamate was lower. Moreover, the levels of aspartate and ornithine, products of glutamate conversion to α-KG by ornithine aminotransferase and glutamate oxaloacetate transaminase, respectively, were increased in allogenic T cells after allo-HCT (2, 31). These data suggest that alloantigen-activated T cells further increase glutaminolysis to replenish intermediate metabolites of the TCA cycle that are depleted in proliferating T cells after allo-HCT. Studies using radioactive tracers indicate that alloreactive CD4^+^ and CD8^+^ T cells preferentially use glutamine to provide substrates for ribose synthesis (31).

### Fatty Acid Metabolism

Alloantigen-activated T cells accumulate various types of FAs and lysophospholipids after allo-HCT (2). In addition to glucose and glutamine, lipids are an effective energy source as well as biosynthetic intermediates (38). FAs can be generated through three different pathways: environmental uptake, synthesis, or hydrolysis of membrane or lipid droplets (39). FAs are classified according to (a) to their backbone lengths (short-, medium-, long-, and very long-chain), (b) saturation, i.e., the number of double bonds (unsaturated, mono-, poly-unsaturated), and (c) position of the double bonds (37). During activation, *in vitro* activated T cells augment fatty acid synthase (FAS) while decreasing FAO, thus enhancing the accumulation of FA metabolites needed for the membrane (17). The effect of lipids on T-cell function seems to be mediated by a complex network dependent on the type of lipids (40).

### Fatty Acid Synthesis

FAs have an important role in Teff function and differentiation. Acetyl-CoA carboxylases 1 (ACC1), ACC2, and FAS are recognized as key rate-limiting enzymes in this process (41). Inhibition of FAS limits development of Th1, Th2, and Th17 subsets (42, 43). Blockade of the enzyme ACC1 enhances the formation of Tregs during Th17 differentiation (43). *In vitro*, induction of FAS after TCR stimulation is regulated *via* the mTORC1–SREBP pathway (14, 44). Moreover, Myc is essential for activation of glucose-metabolizing genes and also for FA synthesis, linking glycolysis to *de novo* FAS (45). Recent studies showed that FAS is required for maintaining glycolytic activity in allogeneic T cells (46). Disruption of FAS at ACC1 effectively ameliorates GVHD development (46, 47). This study emphasizes the relationship between glycolysis and FAS in allogeneic T cells.

### Fatty Acid Oxidation

Fatty acid oxidation is a multistep energetic process by which FAs are broken down in the mitochondria *via* sequential removal of 2-carbon units at the β-carbon position of a fatty acyl-CoA molecule (39, 48). A given long-chain acyl-CoA that enters the FAO yields one molecule of acetyl-CoA from each cycle of FAO. This acetyl-CoA can be directly shuttled into TCA cycle. The NADH and FADH_2_ produced during FAO and the TCA cycle are then available to be used. While saturated short long-chain FA (SCFAs) and medium chain FA are almost exclusively oxidized in the mitochondria, long-chain FA and very long-chain fatty acids (>14 carbons) can also be oxidized in peroxisomes (49). Previous studies have indicated that alloreactive T cells increase FAO, and that targeting FAO could arrest GVHD in haploidentical allo-HCT (8, 9). Although they reported substantial increases in FA transport and intracellular acylcarnitines, suggesting changes in FA metabolism, it was not determined if FAO was directly responsible for the increase in OXPHOS (31, 34). Also, no improvement in survival of recipients treated with FAO inhibitors was shown. By contrast, our recent study showed intracellular carnitine-derived metabolites were diminished in alloantigen-activated T cells after MHC-mismatched or haploidentical allo-HCT (2). Allogeneic T cells dramatically decreased mitochondrial-dependent FAO and pyruvate oxidation through the TCA cycle. Therefore, it is possible that FAO is downregulated in allogeneic T cells after allo-HCT. These inconsistent observations likely result from the different controls used in these two studies. While studies from Ferrara’s group compared bioenergetic parameters of allogeneic T cells to naïve/resting T cells (9), we used those isolated from syngeneic recipients as controls (2); intended to account for homeostatic proliferation of T cells under an inflammatory environment (29). In addition, we observed both Glut1 and Glut3 expression could serve as indicators of glycolytic activity (9), as alloreactive T cells increase Glut3 to an even larger extent than Glut1 in allogeneic recipients (2). Taken together, with study from by Rathmell’s group (24), we speculate that FAO might not be the major material resource fueling the TCA cycle and OXPHOS in alloreactive T cells.

### Sphingolipids (SLs) in Allogeneic T-Cell Metabolism

Sphingolipids represent a major class of lipids important for cell membrane formation (50). S1P is emerging as a key regulator of proliferation, inflammation, vasculogenesis, and resistance to apoptotic cell death (51). Recently, a report demonstrated that S1P1 regulates T cell metabolism through activation of mTOR-Akt, which suppressed Treg function (52). Blockade of the S-1P receptor effectively prevents GVHD by modulating the migration of allogeneic T cells. Ceramide plays a central role in the metabolism of SL (53, 54). Ceramide can be generated *via de novo* synthesis or by degradation of complex SLs, especially sphingomyelin (51). The key rate-limiting step in the biosynthesis of ceramide is the attachment of various acyl-CoA side chains to a sphingoid base by ceramide synthases (CerS) (55). The CerS show substrate preferences for specific chain lengths of fatty acyl CoAs. Briefly, CerS1 shows significant preference for C18-FA CoA, CerS4 for C18-/C20-FA CoA, CerS5 and CerS6 for C16- FA CoA, CerS2 for C22/C24- FA CoA, and CerS3 for ultra-long-chain FA CoA (51, 56). Recent work from our lab showed that CerS6 regulates SL metabolism in alloantigen-activated CD4^+^ and CD8^+^ T cells and required for alloreactive T cells to induce GVHD (57).

## The Role of PD-1 and Check Point Blockade on Allogeneic T Cell Metabolism

The coinhibitory receptor programmed death 1 (PD-1; CD279) has key roles in modulating T-cell responses in both normal and antitumor immunity (58). PD-1 binds to PD-L1 (B7-H1; CD274), which is expressed by macrophages, DCs and non-hematopoietic cells, and PD-L2 (B7-DC; CD273), which is primarily expressed by monocytes and inflammatory macrophages in GVHD target organs (59, 60). Donor T cells significantly upregulate PD-1 expression, which can increase in response to FAO, superoxide, hyperpolarized mitochondrial membrane potential, and ROS formation which subsequently induces T-cell death following allo-HCT. In the absence of PD-1/PD-L1 ligation, donor T cells displayed higher glycolytic activity and OCR. Hence, PD-L1/PD-1 ligation, versus that of PD-L2/PD-1, plays a predominant role in downregulating GVHD (59).

## Microbiota Regulates T Cell Metabolism

The composition, or diversity, of intestinal microbiota shapes the innate and adaptive immune responses (61). The onset of GVHD is associated with a progressive reduction in microbiota diversity, with an increase in Lactobacillales and Blautia and a decrease in Clostridiales species (62–64). The microbiota metabolome, which consists of products generated by host metabolism, microbial metabolism, and mammalian–microbial co-metabolism in the intestines, influences the development of GVHD (65, 66). SCFA-bacterial metabolites, derived from carbohydrate fermentation and include acetate, propionate, isobutyrate, and butyrate, increase histone H3 acetylation in the locus of Foxp3; thereby increasing the numbers of Tregs directly, yet also indirectly through increasing the production of TGFβ in the intestinal epithelium (67). The effect of SCFAs on T cells is also related to mTOR activation (68). SCFAs induce the expression of receptor GPR15, which is responsible for the recruitment of Tregs to the large intestine (69–71). Restoration of butyrate, which is diminished in intestinal epithelial cells (IECs) after allo-HCT, improved IEC junctional integrity, decreased apoptosis, and mitigated GVHD (66). Aryl hydrocarbon receptor (AhR) is a cellular metabolic sensor (72). AhR ligands are derived from intestinal microbiota metabolism. AhR ligand, indole-3-aldehyde, produced by Lactobacilli through tryptophan breakdown (73), modulates the development of GVHD through inducing Tregs and Tr1 cells (74).

## Targeting T-Cell Metabolism to Separate GVHD and the Graft-Versus-Tumor Effect

Given that tumors and alloreactive T cells share a glycolytic phenotype, pharmacological glycolysis inhibition could prevent both GVHD and tumor relapse, a primary complication after allo-HCT. Inhibition of glucose-metabolizing enzymes could reduce allogeneic T activation and function (2, 17) and, further, lower levels of glycolysis would support the generation of long-lived CD8 Tm (3) which are required for maintaining the graft-versus-tumor (GVT) effect. Moreover, *in vivo* activated CD4^+^T cells are more dependent on glycolysis than CD8^+^T cells (75), which are critically important for maintaining GVT activity in allo-HCT. Increasing evidence indicates that CD8^+^ T cells with lower rates of glycolytic activity have better antitumor efficacy in eradicating established tumor in adoptive T cell transfer (ACT) models (76). Blocking glucose metabolism at HK2 by 2-deoxyglucose improves antitumor efficacy of ACT therapy (40). The aforementioned evidence suggests a valid possibility of targeting glycolysis to treat GVHD while preserving the GVT effect after allo-HCT.

## Impact of Current Immunosuppressive Drugs on T-Cell Metabolism in Allo-HCT

Corticosteroids inhibit glycolysis and endogenous respiration in donor lymphocytes and impair GVL activity (77). Inhibiting mTOR with rapamycin decreases glycolysis and enhances FAO in donor T cells; this is expected to reduce alloreactive T cells and enhance Treg function (27). However, attempts to conceptually translate this into patients have proven difficult. This challenge may be because rapamycin can promote CD8 memory T-cell responses by enhancing FAO and hence be detrimental in establishing tolerance (78). Alternatively, inhibition of calcineurin with cyclosporine diminishes glycolytic activity of donor T cells by decreasing glycolytic enzymes and the expression of glut1/3 (79); which support Treg expansion and GVHD attenuation (80).

## Concluding Remarks

Current immunosuppressive regimens, including steroids and calcineurin inhibitors, help to prevent allograft rejection and GVHD. Consequently, patients are vulnerable to complications, such as opportunistic infections and tumor relapse. Therefore, bioenergetic signatures of immune cells at different stages of tolerance induction after transplant could serve as a promising clinical therapeutic strategy. Metabolism inhibitors, in concert with cancer immunotherapies, highlight an avenue by which to achieve better antitumor efficacy and functional tolerance to allografts. Hence, distinguishing metabolic signatures between allogeneic T cells and tumor cells is critical to truly fulfilling this goal.

## Author Contributions

HN and XZ-Y wrote manuscript and HN, SK, DB, and XZ-Y revised manuscript.

## Conflict of Interest Statement

The authors declare that the research was conducted in the absence of any commercial or financial relationships that could be construed as a potential conflict of interest.
